# *Fasciola hepatica* primoinfections and reinfections in sheep drive distinct Th1/Th2/Treg immune responses in liver and hepatic lymph node at early and late stages

**DOI:** 10.1186/s13567-022-01129-7

**Published:** 2023-01-10

**Authors:** María Teresa Ruiz-Campillo, Diana María Barrero-Torres, Nieves Abril, José Pérez, Rafael Zafra, Leandro Buffoni, Álvaro Martínez-Moreno, Francisco Javier Martínez-Moreno, Verónica Molina-Hernández

**Affiliations:** 1 Departamento de Anatomía y Anatomía Patológica Comparadas y Toxicología, Facultad de Veterinaria, UIC Zoonosis y Enfermedades Emergentes ENZOEM, Universidad de Córdoba, Edificio de Sanidad Animal, Campus de Rabanales, Ctra. Madrid-Cádiz Km 396, 14014 Córdoba, Spain; 2 Departamento de Bioquímica y Biología Molecular, Universidad de Córdoba, Edificio Severo Ochoa, Campus de Rabanales, Ctra. Madrid-Cádiz Km 396, 14014 Córdoba, Spain; 3 Departamento de Sanidad Animal (Parasitología), Facultad de Veterinaria, UIC Zoonosis y Enfermedades Emergentes ENZOEM, Universidad de Córdoba, Edificio de Sanidad Animal, Campus de Rabanales, Ctra. Madrid-Cádiz Km 396, 14014 Córdoba, Spain

**Keywords:** *Fasciola hepatica*, primoinfections, reinfections, immune response, liver, hepatic lymph nodes, Th1, Th2, Treg, vaccine

## Abstract

The expression of proinflammatory (IL-1β, IFN-γ, TNF-α) and regulatory (IL-10, TGF-β, IL-4) cytokines, as well as the transcription factor FoxP3, was quantified in the liver and hepatic lymph node (HLN) of sheep primoinfected and reinfected with *Fasciola hepatica* at early (4, 8 and 16 days post-infection [dpi]) and late (100 dpi) stages. The liver exerted a Th2 immune response at very early stages after the primoinfection with *F. hepatica* that induced the downregulation of IFN-γ, followed by a Th1/Th2/Treg response although the late stages were characterised by the expression of Th1/Th2 immune mediators. Contrarily, in reinfected sheep a robust mixed Th1/Th2/Treg immune response was found at very early stages meanwhile at late stages we observed a Th2/Treg immune response overcoming the expression of Th1 immune mediators. However, the HLN displayed a completely different Th1/Th2/Treg expression profile compared to the liver. Primoinfections with *F. hepatica* in HLN induced a mixed Th1/Th2/Treg environment from early stages, establishing a Th2 immune response at a late stage. However, the reinfected sheep exerted a Th2 immune response at early stages led by the IL-4 expression in opposition to the Th1/Th2/Treg found in the liver, meanwhile at late stages the HLN of reinfected sheep exerted a mixed Th1/Th2/Treg immune response. This is the first work publishing the expression of immune mediators in the liver and HLN from reinfected sheep with *F. hepatica.* The study of the immune responses exerted by the natural host in the target organs directly implied in the development of *F. hepatica* are crucial to better understand the immunopathogenesis of the fasciolosis being a key factor to develop effective vaccines.

## Introduction

Fasciolosis, also known as liver fluke disease, is caused by trematodes of the genus Fasciola. *Fasciola hepatica* has one of the greatest geographical distributions for parasites infecting livestock and causes high annual economic losses in many countries [1–3]. In addition, fasciolosis constitutes a zoonosis with a significant economic public health importance all over the world and it is considered by the WHO as a re-emerging neglected tropical disease. Foodborne trematodiasis are most prevalent in Asia and Latin America areas in which basic hygiene measures are not always followed and humans consume raw vegetables or water contaminated with metacercariae [4, 5]. Treatment of fasciolosis is based on the use of chemical drugs with triclabendazole the most commonly used. Nevertheless, its continued and frequent use for livestock treatment has resulted in the emergence of resistance against this drug in *F. hepatica* [6, 7]. In addition, public concern about the presence of drug metabolites in foodstuff is increasing in numerous countries [8]. In this worrying scenario, the search for new formulations, new compounds and re-purposing of drugs as effective treatments of fasciolosis should be strongly considered. In the future vaccines may be an effective therapy against *F. hepatica*. They have been highlighted as the most suitable option since, apart from being sufficient for fluke control, do not show the drawbacks of drug therapy [9–11].

Since the 1990’s numerous vaccination assays against *F. hepatica* have been described in the literature, although only a few of them have been performed in ruminants [5]. The vaccine candidates used in these formulations, either as single molecules or in combination, have shown variable protection levels in ruminants, ranging from 0 to 89.6% [11]. To date, vaccination based on the use of several recombinated antigens (polyvalent vaccines) is showing promising results and good levels of protection [12–15]. Vaccination against *F. hepatica* seems to be suitable, but levels of protection and reliability of the vaccine should be improved. This is related to two main aspects: the characterization of the immune response of the definitive host against the parasite, and the identification of the parasite molecules that could confer a high level of protection in vaccinated animals [5]. Lately, -omics studies in *F. hepatica* have been crucial to discovering molecules as potential vaccine candidates that are implied in the main biological and developmental processes in the parasite, and important to host-parasite interactions. Moreover, a deeper knowledge of the immunopathogenesis of this liver fluke and the molecular interactions between host-parasites in early and late stages of infection might be determinant in developing effective formulations [5, 7].

The ability of *F. hepatica* to establish successful infections is related to the great diversity of molecules with immune regulatory properties that it possesses. This makes chronicity one of the consequences of the regulated milieu induced by the parasite, and the main obstacle in producing an effective vaccine [16, 17]. The capacity of *F. hepatica* to downregulate the Th1 proinflammatory immune response and upregulate the Th2 anti-inflammatory immune response beginning at early stages of infection in sheep has been previously described [18–22]. This scenario implies the downregulation of IFN-γ expression and the upregulation of IL-4 expression, leading to the suppression of the Th1 pro-inflammatory immune response and promoting a Th2 non-protective immune response. This imbalance towards a Th2 immune profile is mediated through regulatory cytokines and cells that modulate and/or suppress inflammatory responses. The induction of a regulatory environment by the expression of cytokines such as IL-10 and TGF-β has been shown as a common strategy used by parasites and microorganisms to extend their survival [23–25]. The expression of FoxP3 regulatory cells is increased as a consequence of this regulatory environment. The expansion in the infected host of FoxP3 facilitating both parasite survival and modulation of tissue damage has been described previously [20, 26–28].

The aim of this study was to evaluate the quantification by qRT-PCR of the expression of pro-inflammatory (IL-1β, IFN-γ, TNF-α) and regulatory (IL-10, TGF-β, IL-4) cytokines, as well as the transcription factor and FoxP3, in the liver and hepatic lymph node (HLN) of sheep primoinfected and reinfected with *F. hepatica* during early and late stages of infection. The quantification of gene expression for proinflammatory and regulatory mediators is essential to understand the molecular, genetic and functional mechanisms of fasciolosis. Thus, a recent study published by our group has established a relationship of reference genes in sheep suitable for infections with *F. hepatica* in the liver and HLN and, in addition, validates other genes related to proinflammatory mediators such as IL-1β and TNF-α, and regulators such as IL-10, TGF-β and FoxP3 [29]. To date, the studies published on gene expression of immunological mediators in fasciolosis include primary infections in ruminants apart from others including reinfections in murine models [30]. Thus, this is the first work including the profile of the immune response in the liver and HLN in sheep reinfected with *F. hepatica*, including early and late stages, which can mimic in some way the natural infection occurring to livestock in the field in comparison to primoinfections.

## Materials and methods

### Experimental design

Fourty-four nine-month old Merino-breed sheep, obtained from a liver fluke-free farm, were used for this study. Animals were tested monthly by parasite egg count technique by faecal sedimentation, with negative results in all cases. The absence of clinical signs for other infectious and/or parasitic diseases was also considered. Four sheep were used as the uninfected control (UC). Fourty sheep were orally infected with a single dose of 200 metacercariae of the Italian strain of *F. hepatica* (Ridgeway Research Ltd, UK) and, subsequently, were divided into two groups (*n* = 20). The reinfected group consisted of twenty sheep that after 9 weeks of administration of a primary oral infection with 200 metacercaries received orally a second dose equal to the first dose. The primoinfected group consisted of twenty orally infected sheep receiving a primary dose with 200 metacercaries in parallell to the second dose of the reinfected group. After the administration of the second dose to the reinfected group and primary dose to the primoinfected group, sheep from both groups were euthanized in batches of 5 animals at 4, 8 and 16 days post-infection (dpi) (early stages) and at 100 dpi (late stage). The size of the groups was calculated according to the magnitude of effect sizes in cytokine expression seen previously [20]. In all animals, the euthanasia was conducted by intravenous injection of 7 mL of Embutramide (200 mg) and Mebezonium iodide (50 mg). No adverse reactions or clinical signs were noted during the experiments. The experiment was approved by the Bioethics Committee of the University of Cordoba (code No. 1118) and conducted in accordance with European (2010/63/UE, Decision 2020/569/UE) and Spanish (L32/2007 and RD 1386/2018) directives on animal experimentation.

### Sample collection and processing

Immediately after euthanasia, sheep were subjected to necropsy and samples from hepatic lymph node (HLN) and left liver lobe were taken. These were washed with diethyl pyrocarbonate (DEPC- Applichem, Panreac, Gatersleben-Germany) biomolecular water, snap frozen in liquid nitrogen and individually disrupted with a mortar and pestle in liquid nitrogen and stored at −80 °C.

### RNA extraction and cDNA synthesis

Total RNA was isolated from 300 mg of grounded tissue (liver or HLN) homogenized in 1.5 mL of TRIzol^®^ reagent (Ambion, life technologies, Carlsbad, CA, USA) using a sterilized IKA^®^T10 basic homogenizer and RNA was extracted with the RNeasy^®^ mini kit (Qiagen, Hilden, Germany) according to the manufacturer’s guidelines. The protocol included a 15 min incubation with RNase-free DNase (Qiagen, Hilden, Germany), and a final 10 min incubation at 65 °C to denature the RNA. RNA purity and concentrations were determined by spectrophotometry. The Agilent 2100 Bioanalyzer (Agilent Technologies, Santa Clara, CA, USA) was used to determine the RNA integrity number (RIN, whose values range from 0 for degraded RNA to 10 for intact RNA. Only RNA with RIN values > 8.5, ratios A_260_/A_280_ about 2 and free of gDNA were used in qRT-PCR experiments.

The cDNA were generated from the 1 μg of total RNA from each sample individually using the iScript™ cDNA Synthesis kit (BioRad, Hercules, CA, USA).

### Primer design

Primers previously designed by our group were used for the quantification of the expression of transcript of the following genes: HSP90, IL-1β, IL-4, IFN-γ, FoxP3, IL-10, TGF-β and TNF-α [20, 29]. All primer pairs produced amplicons of the predicted size (Table 1).

**Table 1:** **Description and sequences of the oligonucleotides designed to quantify specific ovine genes using real-time PCR**.

Genes	Sequences	Amplicon size (bp)	Accession number
HSP90AA1	F 5′-GCCGCCCCTGGAAGGAGACGACGACACG-3′ R 5′-GCCAGGCGAGCCTCGGCAGCGCTCA-3′	130	XM_004017995.3
IL-1β	F 5′-GAAGCTGAGGAGCCGTGCCTACGAACA -3′ R 5′-CCAGCACCAGGGATTTTTGCTCTCTGTCC -3′	185	NM_001009465.2
IL-4	F 5′-CATGTGCTTGAACAAATTCCTGGGCGGAC-3′ R 5′-TAGCCTTTCCAAGAGGTCTCTCAGCGTAC-3′	124	NM_001009313.2
IFN-γ	F 5′-ACCGATTTCAACTACTCCGGCCTAACTC-3′ R 5′-CAGAAAAACCCAAAAGCACACAGAGCAG-3′	97	NM_001009803.1
FoxP3	F 5′-GCCCATCTGGCTGGGAAGATGGCCCAAACC-3′ R 5′- AGAGGTGCCTCCGCACGGCAAACAGG-3′	166	NM_001144947.1′
IL-10	F 5′-TCAGCCGTGCTCTGTTGCCTGGTCTTCC-3′ R 5′- GGACGTCCCGCAGCATGTGGGGCAG-3′	124	NM_001009327.1
TGF-β	F 5′- GGGCTTTCGCCTCAGTGCCCACTGTTC-3′ R 5′- CAGAGGGGTGGCCATGAGGAGCAGG-3′	151	NM_001009400.1
TNF-α	F 5′-CCACGCTCTTCTGCCTGCTGCACTTCGG -3′ R 5′-AACGTGGGCTACCGGCTTGTTATTTGAGGC -3	146	NM_001024860.1

### Absolute quantification of cytokine transcript by real-time PCR

Real-time PCR reactions were performed in triplicate by using 50 ng of cDNA template, 0.3 μM of each primer and the SsoAdvanced™ Universal SYBR^®^ Green Supmermix (BioRad, Hercules, CA, USA) kit, according to the manufacturer’s guidelines. A MyiQ™2 Two Color Real-Time PCR Detection System (BioRad, Hercules, CA, USA) was used. Cycling conditions consisted of 2 min at 95 °C for Platinum Taq activation followed by 40 cycles for melting (15 s, 95 °C), annealing and extension (30 s, 68 °C). After 40 cycles, a melting curve analysis was performed (60–95 °C) to verify the specificity of amplicons. Replicate PCR reactions generated highly reproducible results with SEM < 10% of the mean (<1% for threshold cycle data). All targets amplified with the same optimal PCR efficiency (100%) and high linearity (r > 0.99) in the range of 20 − 2 × 10^5^ pg of total RNA input. An inter-run calibrator (IRC) RNA sample, with a known amount of transcripts of the A170 gene, was introduced in each experiment to guarantee the quality of the retro-transcription and to detect and remove inter-run variation. An absolute calibration curve was constructed, and the number of transcript molecules was calculated from the linear regression of the standard curve.

### Statistical analysis

The software IBM^®^SPSS^®^ statistics version 21.0. was used for the statistical study. The number of mRNA molecules per μg total RNA are shown with averages and standard error of the mean (SEM). Kolmogorov–Smirnov and Saphiro-Will tests were carried out to check whether data distribution is subjected to normal distribution. As data did not show a normal distribution, the non-parametric Mann–Whitney U test was used. A comparison between each infected group (primoinfected or reinfected) and the UC group was carried out. Moreover, a comparison between both primoinfected and reinfected groups in each timepoint was performed. *P* values < 0.01 were considered very statistically significant and *P* values < 0.05 were considered statistically significant.

## Results

### Cytokine gene expression in the liver

**Figure 1:**
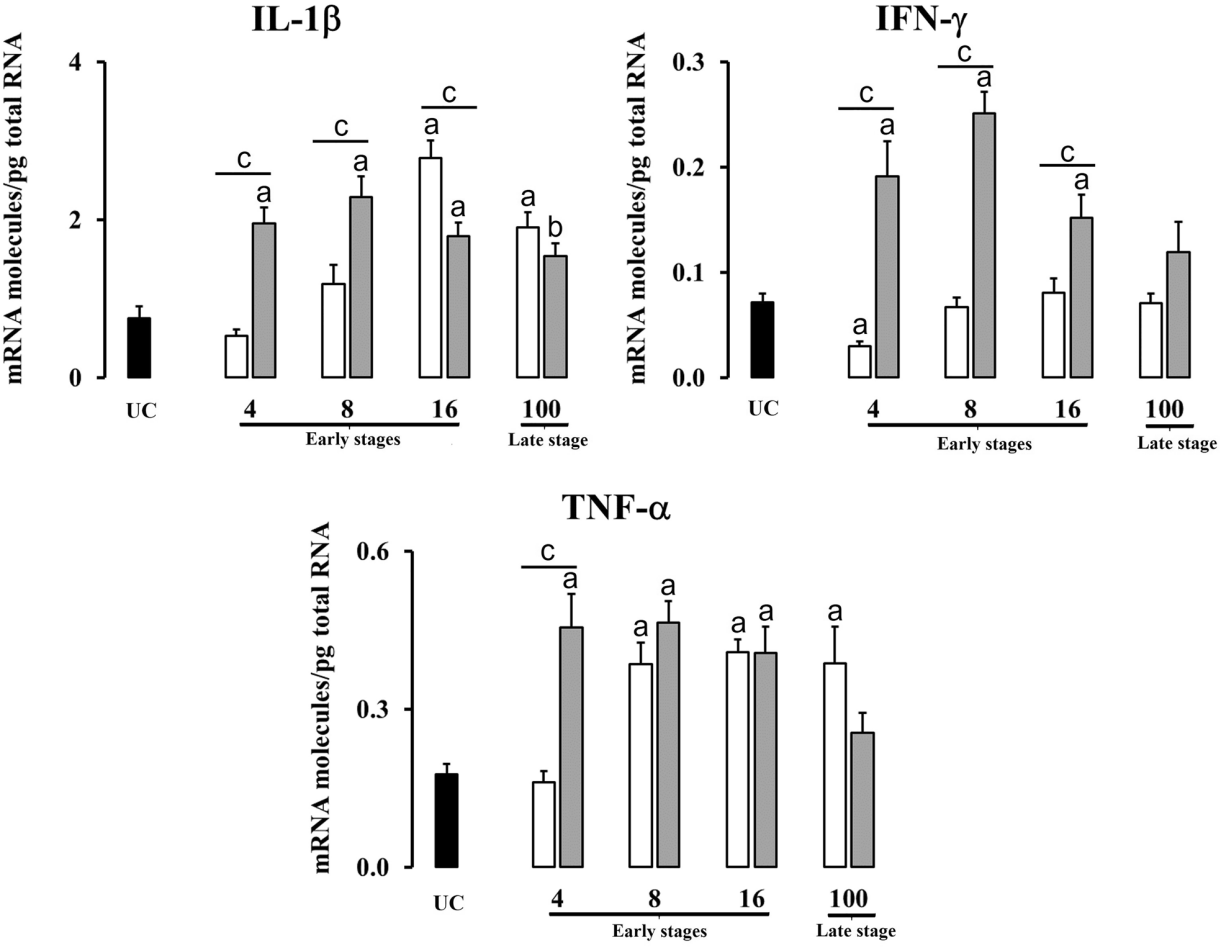
**Gene expression levels in th liver of proinflammatory cytokines (IL-1β, IFN-γ, TNF-α).** Each bar represents the mean ± SEM of the mRNA molecules/pg of total RNA quantified individually in each of the 5 animals per experimental condition and sampling time after three real-time PCR reactions per individual. The non-parametric Mann–Whitney U test was used. A comparison between each infected group (primoinfected and reinfected) and the UC group was carried out showing significant differences: **a** P < 0.01 or **b** P < 0.05. A comparison between the primoinfected and reinfected groups in each timepoint was performed showing significant differences: **c** P < 0.01 or **d** P < 0.05. White bars: primoinfected group; grey bars: reinfected group.

The expression in the liver of pro-inflammatory cytokines (IL-1β, IFN-γ, TNF-α) is shown in Figure 1. The significant increases or decreases of cytokine expression between groups are summarized in Table 2. In primoinfected animals, the expression of IL-1β increased significantly (*P* < 0.01) at 16 dpi and 100 dpi in comparison with the UC. In the reinfected group, this cytokine presents overexpression from 4 to 16 dpi (*P* < 0.01) and at 100 dpi (*P* < 0.05) compared to the UC. In addition, significant differences (*P* < 0.01) were observed between primoinfected and reinfected groups at 4, 8 and 16 dpi. The IFN-γ expression in the primoinfected group shows a significant downregulation (*P* < 0.01) at 4 dpi compared to the UC and no changes were observed in the following timepoints nor early nor late stages. However, the expression of this cytokine in the reinfected group increased significantly (*P* < 0.01) at 4, 8 and 16 dpi with respect to the UC and primoinfected groups. Regarding the TNF-α expression, a significant increase (*P* < 0.01) was observed in the primoinfected group from 8 dpi up to 100 dpi in comparison with the UC, meanwhile in the reinfected group an overexpression (*P* < 0.01) was observed from 4 to 16 dpi with respect to the UC group. Significant differences (*P* < 0.01) between primoinfected and reinfected groups are displayed only at 4 dpi.

**Table 2:** **Significant increases and decreases of cytokine expression between groups at different time-points**.

		Early stages			Late stage
		4 dpi	8 dpi	16 dpi	100 dpi
Liver	PI vs UC	⇊ IFN-γ ↑ IL-4 ↓ FoxP3	⇈ TNF-α; IL-10; TGF-β; IL-4; FoxP3	⇈ IL-1β; TNF-α; IL-10; TGF-β; IL-4; FoxP3	⇈ IL-1β; TNF-α; IL-4
	RI vs UC	⇈ IL-1β; IFN-γ; TNF-α; IL-10; TGF-β; IL-4; FoxP3	⇈ IL-1β; IFN-γ; TNF-α; IL-10; TGF-β; IL-4; FoxP3	⇈ IL-1β; IFN-γ; TNF-α; IL-10; TGF-β; IL-4; FoxP3	⇈ IL-10; TGF-β; IL-4; FoxP3 ↑ IL-1β
	RI vs PI	⇈ IL-1β; IFN-γ; TNF-α; IL-10; TGF-β; IL-4; FoxP3	⇈ IL-1β; IFN-γ; TGF-β ↑ FoxP3	⇈ IFN-γ; IL-4 ↑ TGF-β ⇊ IL-1β	⇈ IL-10; TGF-β
HLN	PI vs UC	⇈ IL-1β; TNF-α; FoxP3 ↑IL-4 ⇊ IFN-γ	⇈ IL-1β; TNF-α; IL-4; FoxP3 ↑ TGF-β ⇊ IL-10 ↓ IFN-γ	⇈ IL-1β; IL-4; FoxP3 ⇊ IFN-γ; IL-10; TGF-β	⇈ IL-4 ⇊ IFN-γ; TNF-α; IL-10; TGF-β; FoxP3
	RI vs UC	⇈ IL-4 ⇊ IFN-γ; TNF-α IL-10; TGF-β ↓ FoxP3	⇈ IL-4 ⇊ IFN-γ; IL-10; TGF-β	⇈ IL-1β; IL-4 ⇊ IFN-γ; TNF-α; IL-10; TGF-β; FoxP3	⇈ IL-1β; TNF-α; TGF-β; FoxP3 ⇊ IFN-γ
	RI vs PI	⇈ IL-4 ⇊ IFN-γ; TNF-α IL-10; TGF-β; FoxP3; ⇂ IL-1β	⇊ IL -1β; IFN-γ; TNF-α; IL-10; TGF-β; FoxP3	⇊ IFN-γ; TNF-α; TGF-β; FoxP3 ↑ IL-4	⇈ IL-1β; IFN-γ; TNF-α; IL-10; TGF-β; FoxP3 ⇊ IL-4

PI: primoinfected group, UC: uninfected group, RI: reinfected group.

⇈ Expression upregulated (*P* < 0.01);

↑ Expression upregulated (*P* < 0.05);

⇊ Expression downregulated (*P* < 0.01);

↓ Expression downregulated (*P* < 0.05).

**Figure 2:**
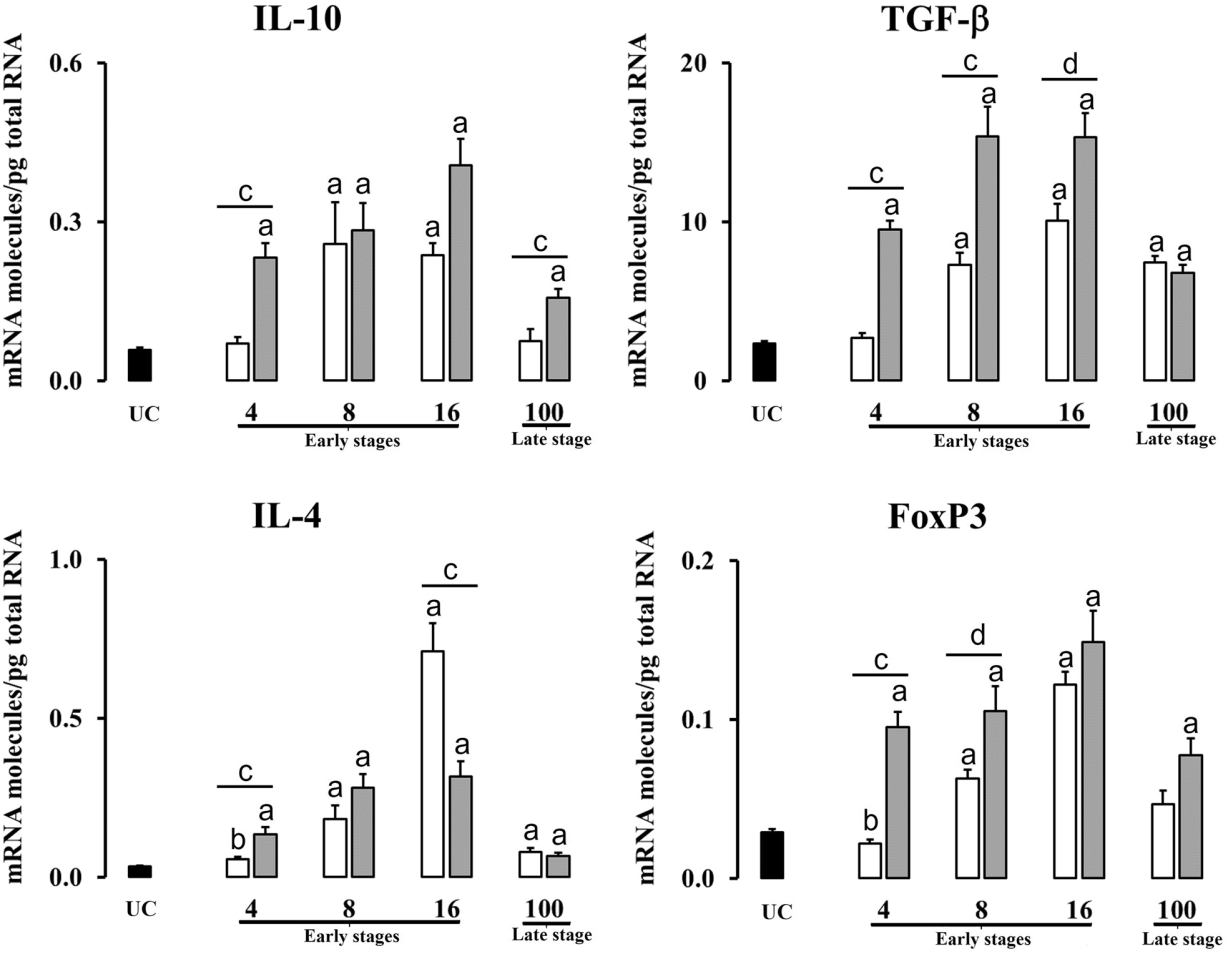
**Gene expression levels in liver of regulatory mediators (IL-10, TGF-β, IL-4 and FoxP3).** Each bar represents the mean ± SEM of the mRNA molecules/pg of total RNA quantified individually in each of the 5 animals per experimental condition and sampling time after three real-time PCR reactions per individual. The non-parametric Mann–Whitney U test was used. A comparison between each infected group (primoinfected and reinfected) and the UC group was carried out showing significant differences: **a** P < 0.01 or **b** P < 0.05. A comparison between the primoinfected and reinfected groups in each timepoint was performed showing significant differences: **c** P < 0.01 or **d** P < 0.05. White bars: primoinfected group; grey bars: reinfected group.

The expression of regulatory cytokines (IL-10, TGF-β, IL-4 and FoxP3) in the liver is shown in Figure 2. The expression of IL-10 increased significantly (*P* < 0.01) at 8 and 16 dpi in the primoinfected group when compared to the UC and no significant differences were found at 100 dpi in comparison with the UC. In the reinfected group, the IL-10 increases significantly (*P* < 0.01) at all timepoints peaking at 16 dpi. However, even when a decrease in IL-10 value at 100 dpi was observed, this remained significantly higher when compared to the UC. The reinfected group also shows a significant increase (*P* < 0.01) with respect to the primoinfected group at 4 and 100 dpi. Regarding the expression of TGF-β, in primoinfected sheep, a significant increase (*P* < 0.01) was observed at 8, 16 and 100 dpi compared to the UC. In reinfected animals, the expression of this cytokine was significantly increased (*P* < 0.01) at all timepoints compared to the UC. In both primoinfected and reinfected groups the gene expression of TGF-β decreased at 100 dpi compared to the rest of the timepoints. However, these values remained significantly higher in comparison with the UC. TGF-β expression was significantly higher in reinfected animals at 4 and 8 dpi (*P* < 0.01) and at 16 dpi (*P* < 0.05) when compared with the primoinfected group. In the primoinfected group, IL-4 increased significantly and gradually from 4 dpi (*P* < 0.05) to 16 dpi peaking at this timepoint, and despite an abrupt decrease displayed at 100 dpi the values remained significatively higher (*P* < 0.01) in comparison with the UC. In the reinfected group, IL-4 showed a significant (*P* < 0.01) overexpression at 4, 8 and 16 dpi compared to the UC. Again, there was a marked decrease at 100 dpi, but still with values significantly higher (*P* < 0.01) compared to the UC. Comparing IL-4 expression between the primoinfected and reinfected groups, at 4 dpi was significantly higher (*P* < 0.01) in the latter, but at 16 dpi IL-4 expression was significantly higher (*P* < 0.01) in the former. The overexpression of FoxP3 in the liver was displayed in primoinfected animals at 8 and 16 dpi (*P* < 0.01), meanwhile at 4 dpi, FoxP3 shows a slight downregulation (*P* < 0.05). In reinfected animals, there was a FoxP3 significant (*P* < 0.01) overexpression in the liver at all timepoints at the early stage. At 100 dpi, only the reinfected group shows significantly higher values compared to the UC. The reinfected group shows significant FoxP3 overexpression compared to the primoinfected one at 4 dpi (*P* < 0.01) and at 8 dpi (*P* < 0.05).

### Cytokine gene expression in hepatic lymph nodes (HLN)

**Figure 3:**
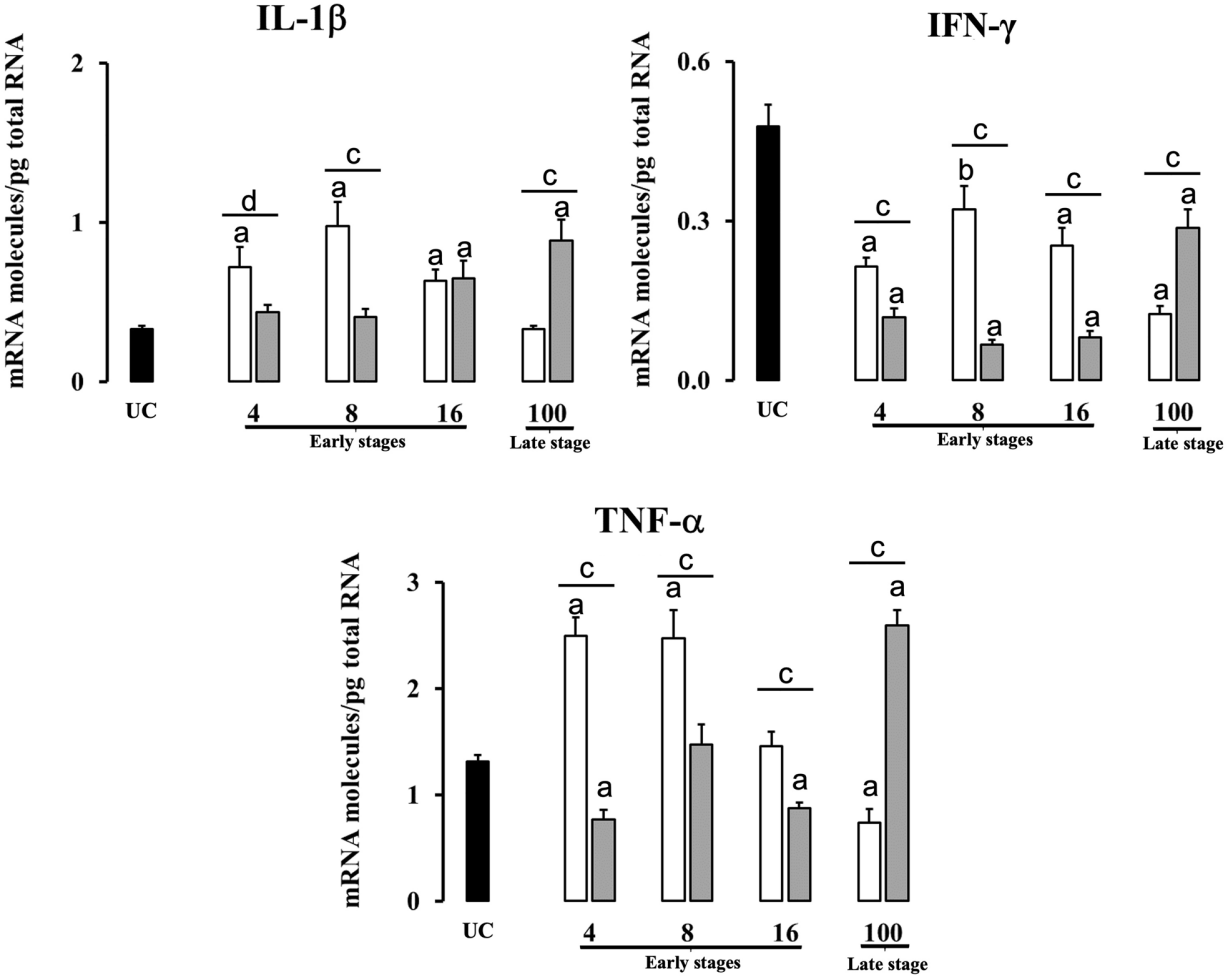
**Gene expression levels in hepatic lymph node of proinflammatory cytokines (IL-1β, IFN-γ, TNF-α).** Each bar represents the mean ± SEM of the mRNA molecules/pg of total RNA quantified individually in each of the 5 animals per experimental condition and sampling time after three real-time PCR reactions per individual. The non-parametric Mann–Whitney U test was used. A comparison between each infected group (primoinfected and reinfected) and the UC group was carried out showing significant differences: **a** P < 0.01 or **b** P < 0.05. A comparison between the primoinfected and reinfected groups in each timepoint was performed showing significant differences: **c** P < 0.01 or **d** P < 0.05. White bars: primoinfected group; grey bars: reinfected group.

Gene expression levels for pro-inflammatory cytokines (IL-1β, IFN-γ, TNF-α) in HLN are shown in Figure 3. The significant increases or decreases of cytokine expression between groups are summarized in Table 2. On the contrary to the liver results, the HLN primoinfected sheep presented a significant (*P* < 0.01) overexpression of IL-1β at 4, 8 and 16 dpi not showing changes at 100 dpi in comparison with the UC. In reinfected animals, IL-1β was significantly (*P* < 0.01) overexpressed at 16 and 100 dpi compared to the UC. The comparison between primoinfected and reinfected groups reveal a significantly higher expression in primoinfected groups at 4 dpi (*P* < 0.05) and 8 dpi (*P* < 0.01), whereas the reinfected groups show higher values at 100 dpi (*P* < 0.01). All primoinfected and reinfected animals at all timepoints show a downregulation of IFN-γ with respect to the UC. This downregulation was particularly severe at 4, 8 and 16 dpi (*P* < 0.01) in the reinfected group, in opposition to the IFN-γ expression displayed in the liver, and at 100 dpi (*P* < 0.01) in the primonfected group. There were statistical differences (*P* < 0.01) between primoinfected and reinfected groups at all timepoints. Regarding TNF-α expression in HLN, a significant overexpression (*P* < 0.01) occurred in primoinfected sheep at 4 and 8 dpi with respect to the UC group. However, at 100 dpi a marked significant downregulation (*P* < 0.01) was observed in the primoinfected group with respect to the UC group. Conversely, reinfected animals show a significant decrease (*P* < 0.01) at 4 and 16 dpi and a significant overexpression (*P* < 0.01) at 100 dpi compared to the UC. These results are contrary to the TNF-α expression in the liver. A comparison between primoinfected and reinfected groups revealed an overexpression in primoinfected sheep at 4, 8 and 16 dpi (*P* < 0.01), whereas at 100 dpi the expression was significantly higher (*P* < 0.01) in the reinfected group.

**Figure 4:**
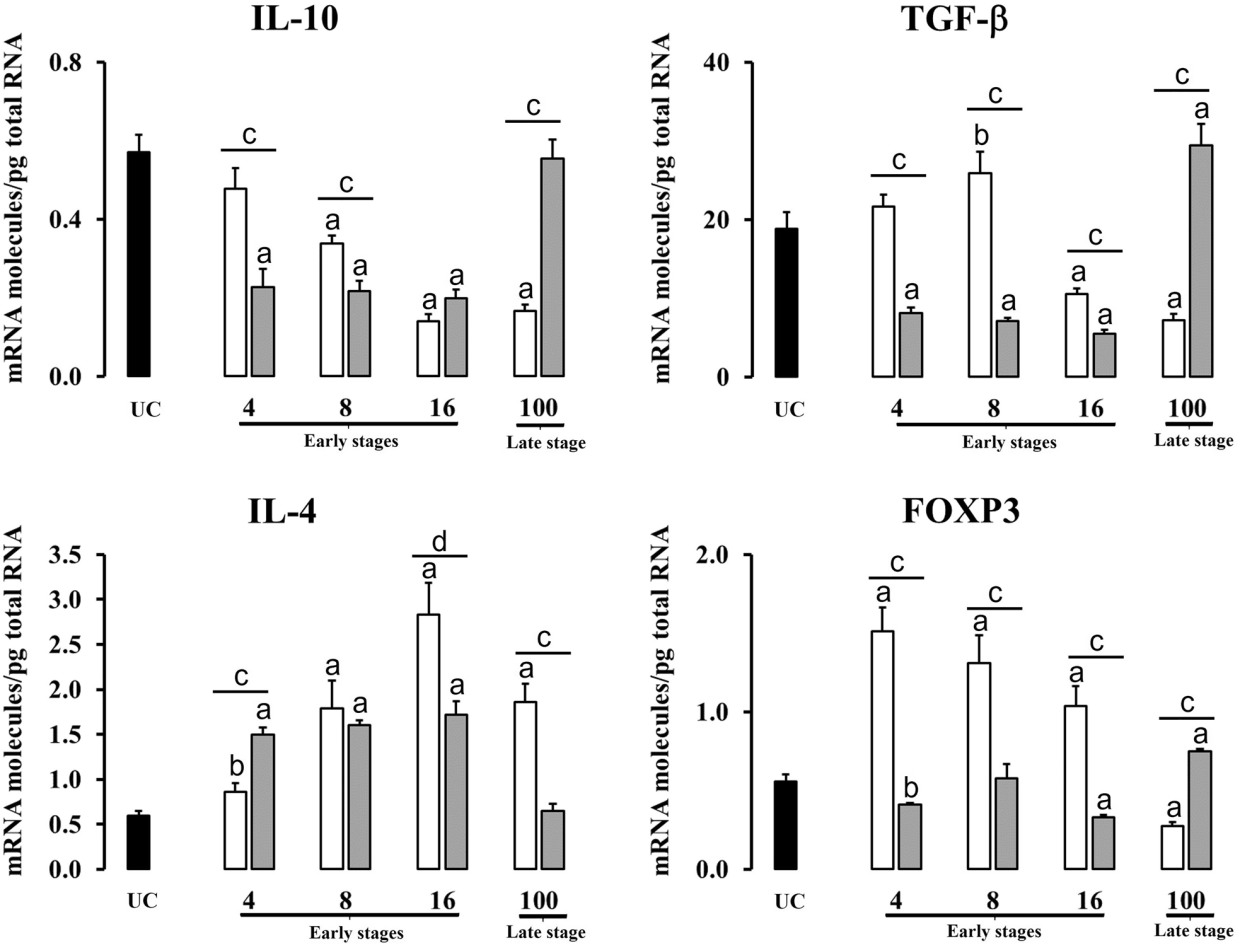
**Gene expression levels in hepatic lymph node of regulatory mediators (IL-10, TGF-β, IL-4 and FoxP3).** Each bar represents the mean ± SEM of the mRNA molecules/pg of total RNA quantified individually in each of the 5 animals per experimental condition and sampling time after three real-time PCR reactions per individual. The non-parametric Mann–Whitney U test was used. A comparison between each infected group (primoinfected and reinfected) and the UC group was carried out showing significative differences: **a** P < 0.01 or **b** P < 0.05. A comparison between the primoinfected and reinfected groups in each timepoint was performed showing significant differences: **c** P < 0.01 or **d** P < 0.05. White bars: primoinfected group; grey bars: reinfected group.

The expression in HLN of regulatory cytokines (IL-10, TGF-β, IL-4 and FoxP3) is shown in Figure 4. In the primoinfected group IL-10 levels show a gradual and significant downregulation (*P* < 0.01) from 8 dpi onwards, whereas reinfected sheep presented a significant reduction (*P* < 0.01) with respect to UC at 4, 8 and 16 dpi. The primoinfected group shows a significant reduction (*P* < 0.01) at 100 dpi with respect to the reinfected ones, meanwhile the reinfected group shows a significant reduction (*P* < 0.01) in the expression of this cytokine at 4 and 8 dpi with respect to the primoinfected sheep. Regarding TGF-β, despite the fact that an overexpression (*P* < 0.05) occurred in the primoinfected group at 8 dpi, a significant decrease (*P* < 0.01) was observed at 16 and 100 dpi with respect to the UC, in opposition to the results found in the liver. Moreover, a significant downregulation (*P* < 0.01) of this cytokine was recorded in reinfected sheep from 4 to 16 dpi on the contrary to the results in the liver, meanwhile a significant overexpression (*P* < 0.01) was found at 100 dpi with respect to UC animals. The comparison between primoinfected and reinfected groups revealed a significantly higher (*P* < 0.01) expression of TGF-β at 4, 8 and 16 dpi in primoinfected animals with respect to reinfected ones, while at 100 dpi the expression of this cytokine was significantly higher (*P* < 0.01) in the reinfected group with respect to the primoinfected one. The expression of IL-4 shows a gradual and significant increase during early stages of infection (4, 8 and 16 dpi) both in the primoinfected and reinfected groups with respect to the UC group similarly to the liver, while at 100 dpi only the primoinfected animals maintained the overexpression (*P* < 0.01) of this cytokine with respect to the UC ones. A comparison between the primoinfected and reinfected groups revealed higher IL-4 expression at 4 dpi (*P* < 0.01) in the reinfected group while overexpression was recorded in the primoinfected group at 16 dpi (*P* < 0.05) and 100 dpi (*P* < 0.01). FoxP3 expression significantly increased (*P* < 0.01) at 4, 8 and 16 dpi in primoinfected animals with respect to the UC ones, however, a significant decrease (*P* < 0.01) was found at 100 dpi in comparison with the UC. In reinfected animals, a significant reduction in FoxP3 values was recorded at 4 dpi (*P* < 0.05) and at 16 dpi (*P* < 0.01) on the contrary to the liver results, but a significant increase (*P* < 0.01) was reported at 100 dpi with respect to the UC animals. A comparison between the primoinfected and reinfected groups revealed overexpression (*P* < 0.01) of this cytokine during early stages of infection (4, 8 and 16 dpi) in primoinfected animals, while at 100 dpi overexpression (*P* < 0.01) occurred in reinfected animals.

## Discussion

This work aimed at quantifying Th1, Th2 and Treg cytokines expressed after primoinfections and reinfections with *F. hepatica* in sheep focusing on the liver as the target organ in the development of juvenile and adult stages, and the hepatic lymph nodes (HLN) as the regional lymphatic organ directly implicated in the hepatic immune response. Other studies have evaluated the expression of proinflammatory and regulatory cytokines in sheep primoinfected with *F. hepatica* [18, 20, 28] and analysed the immune profile through the gene expression of Th1/Th2/Th17/Treg cytokines in reinfected rats [30]. This is the first study, however, quantifying the gene expression profile of Th1/Th2/Treg cytokines of reinfected sheep in both liver and HLN.

The primoinfection in the liver drove the establishment of a Th2 immune response by the expression of IL-4 at 4 dpi, meanwhile FoxP3 was downregulated at this earlier stage. Moreover, a lack of Th1 immune response against *F. hepatica* infection was observed at this time-point associated with the IFN-γ downregulation induced by *F. hepatica*. Many studies indicate that the immunosuppression exerted by *F. hepatica* in in vivo and in vitro models is by due to the downregulation of the Th1 immune response by the suppression of IFN-γ expression [31–34]. However, few of them have focused on the very early stages of *F. hepatica* infection in sheep [18, 20] and Pacheco et al. reported no significant changes in the gene expression of IFN-γ in the livers of sheep infected with *F. hepatica* until 18 dpi. The early immunomodulation towards a Th2 immune response induced by *F. hepatica* overcoming a Th1 response seems to be the point of no return to allow the entry and migration of the parasite within the liver parenchyma causing severe liver damage. Moreover, it has been claimed as a mechanism of survival of the parasite within the host in contrast to the proinflammatory environment which results in parasite clearance [9, 21, 35, 36]. From 8 dpi, a mixed Th1/Th2/Treg response was predominantly induced by the parasite in primoinfected sheep highlighting the absence of IFN-γ expression changes, meanwhile the rest of proinflammatory and regulatory cytokines and the transcription factor FoxP3 were upregulated during the early stage of infection. This Th1/Th2/Treg environment has been previously reported by other authors at early stages of infections with *F. hepatica* [20, 28, 37, 38]. The increase of FoxP3 expression associated with the expansion of Tregs at early stages are consistent with the results found in goats and sheep infected with *F. hepatica* or *Teladorsagia circumcincta* [20, 28, 39, 40].

However, the immune response in the liver at late stages of the primary infection exerted a mixed Th1/Th2 response including the participation of cytokines such as IL-1β, TNF-α, TGF-β and IL-4 as it has been reported during acute and chronic stages in ruminants and other species [18, 31, 41, 42]. The absence of the Tregs, as well as IL-10 at this later stage could be responsible for the severe hepatic damage caused by *F. hepatica*. On the contrary to the early Th2 *F. hepatica*-induced immune response in the liver after primary infections, in reinfected sheep, a robust mixed Th1/Th2/Treg immune response was observed from 4 dpi and established along the early stage of infection being characterised by the expression of proinflammatory cytokines (IL-1β, IFN-γ and TNF-α) and regulatory mediators (IL-10, TGF-β, IL-4 and FoxP3). The mixed Th1/Th2/Treg immune response exerted in the very early stages of reinfections can be the result of a previous exposure to *F. hepatica* inducing an earlier proinflammatory and regulatory immune response. Interestingly, IFN-γ was upregulated along the early stage of reinfection in contrast to the results found after primoinfections. It has been suggested that the increase of IFN-γ in the liver of sheep after *F. hepatica* infections may reflect a response to tissue damage and granuloma formation rather than a response to the parasitic larvae [18]. Since in reinfections, adult worms cause hepatic damage during feeding, it is possible that the higher levels of IFN-γ observed in reinfected groups may be a response to tissue damage caused by both adult and migrating flukes. Previous works have suggested that reinfections with *F. hepatica* or *F. gigantica* fail to stimulate any resistance or sensitization in sheep on the contrary to other species such as cattle and rats although the results were based on parasitological and haematological parameters, hepatic enzymatic assays, and proliferative and antibody responses [43, 44]. In this study, we show a robust mixed Th1/Th2/Treg immune response in reinfections by quantifying the expression of proinflammatory and regulatory cytokines, as well as the transcription factor FoxP3. Despite the promotion of a regulatory response, the parasite induces a proinflammatory immune response to avoid an exacerbated Th2 response that could be deleterious for the parasite [14, 45]. Similarly, the intestinal parasite *Heligmosomoides polygyrusbakeri* induces the IL-1β secretion which in turn inhibits IL-33 and IL-25, resulting in the control of an excessive Th2 response [46]. Thus, the presence of Tregs associated to the upregulation of IL-10 in reinfections with *F. hepatica* confirms the ability of the parasite to modulate immune responses facilitating its survival. It is therefore an important cell type with immunomodulator effect in helminth infections [27, 47]. The overexpression of TGF-β in reinfected sheep at early stages of infection compared to the primoinfected ones could be related to the fibrosis process that takes part in the liver with chronic lesions [20, 38, 48].

However, at late stages of reinfection, the Th2/Treg immune response orchestrated by the parasite was more robust, overcoming the Th1 immune response, displaying only upregulation of the IL-1β in contrast to the overexpression exerted by all the regulatory mediators studied. These results were consistent with those found in reinfections at chronic stages in rodent models [30, 49], although in our study the expansion of Tregs and expression of IL-10 are dependent on the secondary infection in the liver of sheep at chronic stages.

The expression of proinflammatory and regulatory cytokines in the HLN displayed a different profile with respect to that exerted in the liver. A study carried out by Meeusen and Brandon, 1994 [50] in rats showed that the antibody secreting cells only recognize antigens from the parasite stage present in the organ drained by the lymph node highlighting the importance of the study of the immune response developed in the HLN of sheep after *F. hepatica* infection in early and late stages of primo- and re-infections. In the HLN of primoinfected sheep, Th1 related cytokines (IL-1β and TNF-α) were overexpressed at early stages of infection, while in reinfected sheep these two cytokines were highly expressed in late stages. The overexpression of these two cytokines in reinfected sheep at late stages could be related to the induction of a proinflammatory immune response to prevent the establishment of an exacerbated Th2 response that could be deleterious for the parasite. IFN-γ was downregulated along the study at early and late stages. The IFN-γ downregulation in HLN was previously reported at early and late stages of *F. hepatica* infections [18, 51]. The suppression of IFN-γ levels in both the liver and HLN indicate the ability of *F. hepatica* to modulate the secretion of this cytokine. This downregulation was also observed in IL-10 in early stages in primo- and reinfected sheep and in TGF-β in reinfected sheep. However, there is an upregulation of TGF-β at late stages in reinfected sheep. Regulatory mediators IL-4 and the transcription factor FoxP3 were expressed from 4 dpi onwards, providing the primoinfections in HLN with a mixed Th1/Th2/Treg environment from 4 dpi onwards. However, on the contrary to the liver results, IL-4 played a predominant role establishing a Th2 immune response at late stages of the primoinfections with *F. hepatica* in the HLN coinciding with the results of Pacheco et al. [18]. At this location, regulatory immune mediators such as IL-10, TGF-β and FoxP3 were downregulated at late stages by the primary infection with *F. hepatica*. These results confirm in sheep the same chronic pattern published previously in bovine fasciolosis by Sachdev et al. [47] whereby Th2 cytokine responses were diminished by week 13 post-infection despite the parasites residing in the liver, being induced by IL-4 in HLN of chronic primoinfected sheep.

Moreover, the HLN in the reinfected sheep exerted a Th2 immune response at early stages led by the IL-4 expression in opposition to the Th1/Th2/Treg found in the liver. Even IFN-γ was downregulated in both the primoinfected and reinfected groups in contrast with the overexpression found in the liver of the reinfected sheep. This result is consistent with the decrease observed in IFN-γ gene expression levels from 1 dpi in HLN described by Pacheco et al. [18].

Conversely to the liver and the results obtained from HLN in primoinfected sheep, in the reinfected group the early stages arweree characterised by a Th2 immune response with the participation exclusively of IL-4. The other proinflammatory and regulatory cytokines studied, as well as transcription factor FoxP3, were downregulated. In the late stages of reinfected sheep, the HLN presented a mixed Th1/Th2/Treg immune response led by the expression of TGF-β, FoxP3, TNF-α and IL-1β. This is the first work published on the immune response in the HLN of sheep reinfected with *F. hepatica* at acute and chronic stages.

In conclusion, the results of the present study revealed several relevant differences in cytokine expression in primoinfected and reinfected sheep, such as the more rapid and severe increase of IL-4 and FoxP3 in the liver and more severe decrease of IFN-γ in HLN of reinfected versus primoinfected sheep. These changes in reinfected sheep should be considered as mechanisms of immunomodulation that may facilitate parasite survival and it may be a reason why the development of protective vaccines for fasciolosis is so difficult. The greater number of metacercariae administered in two doses to the reinfected sheep could, however, play a role in the differential immune response at very early stages of infection in comparison to the primoinfections. Since natural *F. hepatica* infection occurs in small repetitive doses during grazing (trickle infection) the use of reinfected animals may be more useful for the study of immunological responses to *F. hepatica* than primoinfected animals that have been used in the majority of studies to date.

## Data Availability

The data presented in this study are available on request from the corresponding author.

## Abbreviations

*F. hepatica*: *Fasciola hepatica*
HLN: hepatic lymph nodes
IL-1β: interleukin-1β
IFN-γ: gamma interferon
TNF-α: tumor necrosis factor-alpha
IL-10: interleukin 10
TGF-β: transforming growth factor-β
IL-4: interleukin 4
FoxP3: Forkhead box P3
qRT-PCR: Real-Time Quantitative Reverse Transcription PCR
RNA: ribonucleinc acid
cDNA: complementary deoxyribonucleic acid
Th1: T helper Type 1
Th2: T helper Type 2
Treg: regulatory T cells
RIN: RNA integrity number
UC: uninfected control
